# Inequalities in cancer screening participation: examining differences in perceived benefits and barriers

**DOI:** 10.1002/pon.4195

**Published:** 2016-07-14

**Authors:** S.G. Smith, L.M. McGregor, R. Raine, J. Wardle, C. von Wagner, K.A. Robb

**Affiliations:** ^1^ Centre for Cancer Prevention, Wolfson Institute of Preventive MedicineQueen Mary University of LondonLondonUK; ^2^ Department of Epidemiology and Public HealthUniversity College LondonLondonUK; ^3^ Department of Applied Health ResearchUniversity College LondonLondonUK; ^4^ Institute of Health and WellbeingUniversity of GlasgowGlasgowUK

**Keywords:** cancer, colorectal cancer screening, education, inequalities, oncology, uptake

## Abstract

**Objective:**

Inequalities exist in colorectal cancer (CRC) screening uptake, with people from lower socioeconomic status backgrounds less likely to participate. Identifying the facilitators and barriers to screening uptake is important to addressing screening disparities. We pooled data from 2 trials to examine educational differences in psychological constructs related to guaiac fecal occult blood testing.

**Methods:**

Patients (n = 8576) registered at 7 general practices in England, within 15 years of the eligible age range for screening (45‐59.5 years), were invited to complete a questionnaire. Measures included perceived barriers (emotional and practical) and benefits of screening, screening intentions, and participant characteristics including education.

**Results:**

After data pooling, 2181 responses were included. People with high school education or no formal education reported higher emotional and practical barriers and were less likely to definitely intend to participate in screening, compared with university graduates in analyses controlling for study arm and participant characteristics. The belief that one would worry more about CRC after screening and concerns about tempting fate were strongly negatively associated with education. In a model including education and participant characteristics, respondents with low emotional barriers, low practical barriers, and high perceived benefits were more likely to definitely intend to take part in screening.

**Conclusions:**

In this analysis of adults approaching the CRC screening age, there was a consistent effect of education on perceived barriers toward guaiac fecal occult blood testing, which could affect screening decision making. Interventions should target specific barriers to reduce educational disparities in screening uptake and avoid exacerbating inequalities in CRC mortality.

## 1 Background

Colorectal cancer (CRC) is a leading cause of cancer death worldwide.^1^ Approximately 40 000 cases of CRC are diagnosed each year in the United Kingdom, making it the third most common cancer.^2^ In England, the National Health Service Bowel Cancer Screening Programme (NHS BCSP) offers once‐only flexible sigmoidoscopy at age 55 years and biennial guaiac fecal occult blood testing (gFOBt) from ages 60 to 74 years. Both modalities reduce CRC mortality.^3^, ^4^

However, participation varies. Data from the first 2.6 million invitations to the NHS BCSP showed an overall uptake of 54%, ranging from 35% in the most deprived neighborhoods to 61% in the least deprived.^5^ The linear association highlights that inequalities in participation are not just between the most and least deprived groups but rather there is a socially graded relationship in uptake.^6^ Individual markers of socioeconomic status such as education, income, and health literacy have been linked to CRC screening uptake in English population–based cohort studies.^7^, ^8^ Similar observations have been made in the US Behavioral Risk Factor Surveillance System survey.^9^ Inequalities in screening participation raise the specter of increasing disparities in CRC mortality.^10^

In the United Kingdom, CRC screening is part of the NHS, and so inability to pay does not explain inequalities in uptake. Behavioral science has made progress in understanding screening behavior by identifying modifiable psychological constructs associated with uptake, eg, perceived benefits and barriers.^10^, ^11^ A theoretical framework has been developed suggesting pathways through which socioeconomic status can influence screening uptake.^12^ A key corollary of the framework is education, which is the focus of the present analysis. The model suggests education is strongly linked with health literacy, a lack of which can lead to negative expectations and beliefs about screening.^13^, ^14^ Such beliefs may remain unchallenged, as people with less education are also less likely to seek information about cancer,^15^ leading to greater uncertainty and anxiety about the disease.^16^, ^17^, ^18^

A consistent body of behavioral science literature has shown that people with more negative expectations and beliefs, and greater anxiety about a behavior are less likely to engage with it.^10^, ^11^, ^12^ However, few studies have sought to identify differences in these factors by educational achievement.^12^ A questionnaire study (n = 1808) in 2 primary care practices in England reported that respondents with less formal education were more worried about cancer than those with more years in education.^17^ A similar UK study based in primary care (n = 964) observed that people with low numeracy were more likely to report emotional (eg, disgust and worry) and practical barriers (eg, privacy concerns) to screening.^19^ Identification of the psychological factors underpinning inequalities in screening uptake can improve behavioral interventions in the area.

The present analyses explored whether there was an educational gradient in perceived benefits and barriers within the context of an established CRC screening program in the United Kingdom (ie, gFOBt sent to 60‐74 year olds every 2 years). Education is used here as a marker of socioeconomic status.^20^ Neighborhood measures of socioeconomic status were not used because they are composed of area‐level markers that we assessed more accurately at an individual level.^21^ Using individual markers of socioeconomic status reduces the risk of misclassification. Education has been shown to explain similar amounts of variance in health behavior outcomes to occupation and income.^22^ In addition, education is a key pathway hypothesized to explain socioeconomic inequalities in screening uptake in our conceptual framework.^12^ We hypothesized there would be a graded association between the outcome measures and education such that participants with less education would report more barriers to gFOBt, fewer benefits, and weaker screening intentions.

## 2 Methods

### 2.1 Procedure

Data were from 2 randomized trials testing narrative and low literacy (“gist‐based”) CRC screening information materials.^23^, ^24^ Patients were from 7 general practices in areas of mixed socioeconomic deprivation in England (narrative = 3; gist = 4). A similar protocol was followed for both trials, allowing the data to be combined. In both trials, a list of patients aged 45 to 59.5 years, which is the age range approaching the eligible age (60 years) for gFOBt screening in England, was created at each practice. Staff excluded patients who had severe cognitive impairment, had a recent significant illness, were under CRC surveillance, or did not speak English. All patients meeting the eligibility criteria in the trials (n = 8576) were sent a study pack containing the information materials used in the NHS BCSP, a questionnaire, and a prepaid envelope. A reminder pack was sent after 4 weeks. A supplementary information leaflet (“gist” or “narrative”) was also included in the intervention groups. The type of leaflet received was the main difference in study design. For these analyses, data were combined to create a single respondent pool. Study group allocation (intervention vs control) was controlled in multivariable analyses. Ethical approval was given in February 2012 (12/NE/0058; 12/YH/0106). Data were collected from June 2012 to January 2013.

### 2.2 Measures

#### 2.2.1 Participant characteristics

Questionnaire items assessed gender, age, marital status, ethnicity, employment status, self‐rated health, and education.

#### 2.2.2 Intention

Intention to be screened for CRC was assessed with a single item, “Imagine you have just turned 60 and have received the bowel screening test kit (FOB test kit) in the post, would you do the test?” Response options were “definitely not,” “probably not,” “yes, probably,” and “yes, definitely.” The item source can be found in the Supporting Information.

#### 2.2.3 Perceived barriers

Five questions assessed perceived emotional barriers toward gFOBt screening (Supporting Information). Response options were on a 4‐point Likert scale (strongly disagree to strongly agree). Score range was 5 to 20, with higher scores indicating stronger endorsement. Internal consistency was adequate (α = .67)*.* Three questions assessed perceived practical barriers to FOBt screening (Supporting Information). Score range was 3 to 12, with higher scores indicating stronger endorsement. Internal consistency was adequate (α = .76).

#### 2.2.4 Perceived benefits

Five items assessed perceived benefits of gFOBt screening (Supporting Information). Responses were on a scale of 1 to 4 (strongly agree to strongly disagree). Score range was 5 to 20, with higher scores indicating stronger endorsement. Internal consistency was adequate (α = .79).

### 2.3 Statistical power

Sensitivity power calculations assuming α = 0.05, power = 0.90, and 3 education groups, suggest a sample of 2104 (the smallest sample in these analyses), would detect a small effect size (odds ratio [OR] = 1.2).

### 2.4 Statistical analysis

Analyses comparing the gender, age, and deprivation of respondents and nonrespondents were completed using χ^2^ and *t* tests. Neighborhood deprivation was assessed by the Index of Multiple Deprivation rank score using home postcodes.^21^ The perceived benefits and barriers scales were described using means. For descriptive purposes, individual items on the scales were categorized as “agree” vs “disagree” and compared across education groups. These analyses were not tested statistically to prevent an inflated type I error. Perceived barriers, benefits, and intention were dichotomized into high and low groups using the median split technique in preparation for a univariate χ^2^ analysis to test differences across educational groups. Multivariable logistic regression controlling for study group (intervention vs control), age, gender, marital status, ethnicity, and self‐rated health was used to investigate the association between education and the outcomes of perceived benefits, barriers, and intention. Pearson's correlation investigated the associations between perceived benefits, barriers, and screening intention. A type I error rate of *P* < .05 was used throughout. Missing data were <2% for all variables. For the perceived barriers and benefits outcomes, data were prorated to account for the number of items responded to. Participants were included in this transformation if they responded to ≥50% of items in the scales (emotional barriers [3 items; n = 2166]; practical barriers [2 items; n = 2163]; perceived benefits [3 items; n = 2169]). Remaining missing data were deleted pairwise. SPSS v 21 was used for analyses.

## 3 Results

In total, 8576 people were sent an invitation to participate, and 6666 were sent a reminder. One hundred six were returned undelivered. Questionnaires were returned by 2860 individuals, of which 2250 were at least partially completed. Questionnaire data on age and gender were compared with practice records, and 69 people were excluded because of discrepancies. The sample for analysis was therefore n = 2181. The cooperation rate was 26.0%.^25^

Nonresponders were more likely than responders to be male (53.8% vs 45.9%, *P* < .001), younger (mean [M] = 50.9 years, standard deviation [SD] = 4.1 vs M = 51.8 years, SD = 4.2, *P* < .001), and from a socioeconomically deprived neighborhood (M = 37.9, SD = 21.5 vs M = 30.4, SD = 20.3, *P* < .001).

Participant characteristics are described in Table 1. The sample was evenly balanced with regard to gender and age. The majority of respondents were married, white, employed, and had a good level of self‐reported health. Over half had a high school or equivalent education (54.2%), with the remaining respondents reporting no formal education (13.7%) or a university‐level education (32.1%).

**Table 1:** Participant characteristics

	**n (%)**
Gender	
Male	999 (45.9)
Female	1177 (54.1)
Age (y)	
45‐49	731 (33.6)
50‐54	746 (34.3)
55‐59	696 (32.0)
Marital status	
Married	1470 (67.7)
Unmarried	700 (32.3)
Ethnicity	
White	1856 (85.6)
Black	110 (5.1)
South‐Asian	86 (4.0)
Other	115 (5.3)
Employment	
Employed	1625 (75.5)
Unemployed	160 (7.4)
Homemaker	104 (4.8)
Retired	72 (3.3)
Student	12 (0.6)
Disabled	178 (8.3)
Self‐rated health	
Poor	129 (5.9)
Fair	533 (24.6)
Good	1204 (55.5)
Excellent	303 (14.0)
Study group	
Narrative information	629 (28.8)
Gist information	498 (22.8)
Standard information	1054 (48.3)
Education	
No formal education	294 (13.7)
High school or equivalent	1160 (54.2)
University graduate	687 (32.1)

The n may not round to 2185 because of missing data.

### 3.1 Emotional barriers

Most emotional barriers were endorsed by less than a fifth of the sample (Table 2). A gradient in the likelihood of agreeing between the lowest and highest education groups could be seen for the items on embarrassment, tempting fate, and worry. A small reverse gradient was observed for the item on disgust (Table 2).

**Table 2:** The likelihood of agreeing/strongly agreeing with perceived barriers, benefits, and intention by educational group

	**Sample (%)**	**No Formal Education (%)**	**High School (%)**	**University Graduate (%)**	**Range**
Emotional barriers					
Doing the FOB test would be disgusting	16.6	15.6	16.0	18.1	−2.5
I would be embarrassed if others knew I had done the FOB test	6.9	10.0	7.4	4.7	5.3
Doing the FOB test would make me worry more about bowel cancer	16.8	26.1	16.2	13.7	12.4
I would be afraid of getting an abnormal result from my FOB test	51.6	55.6	51.2	50.5	5.1
Doing the FOB test would be tempting fate	6.4	15.7	5.4	4.1	11.6
Practical barriers					
I would not want to keep small amounts of my stools on a card in the house	14.2	17.6	14.7	11.7	5.9
I would not have the privacy to do the FOB test	4.4	9.0	4.0	3.1	5.9
I would be unlikely to have the time to do the FOB test	4.2	9.1	3.2	3.8	5.3
Perceived benefits					
Doing the FOB test would be an important thing for me to do	94.7	93.8	94.8	95.0	−1.2
Doing the FOB test would make me feel I was doing something positive for my health	96.8	94.5	97.4	96.6	−2.1
Doing the FOB test would give me peace of mind	92.7	92.5	93.9	90.7	1.8
Doing the FOB test and receiving a normal result would reassure me that I do not have bowel cancer	90.3	92.4	91.1	87.9	4.5
I would do the FOB test because I would want to stay healthy for my family	93.0	92.4	93.4	92.4	0
Intention (definitely)	74.2	65.9	73.4	79.1	−13.2

Abbreviation: FOB, fecal occult blood.

The n for each educational group varies because of missing data. Emotional barriers: no educational qualifications, n = 284‐291; some formal qualifications, n = 1132‐1151; university qualifications, n = 682‐686; practical barriers: no educational qualifications, n = 287‐290; some formal qualifications, n = 1147‐1151; university qualifications, n = 682‐686. Benefits: no educational qualifications, n = 289‐292; some formal qualifications, n = 1146‐1156; university qualifications, n = 679‐685; intention: no educational qualifications, n = 293; some formal qualifications, n = 1155; university qualifications, n = 685.

The scale mean was 9.52 (SD = 2.36) of 20, indicating low to moderate agreement. The likelihood of experiencing high emotional barriers increased across the education categories (χ^2^[2] = 36.14, *P* < .001). Over two‐thirds (68.0%) of those with no formal education experienced high emotional barriers, compared with 55.3% and 47.3% in the high school or equivalent and university graduate education categories, respectively. In multivariable analysis, compared with the university‐educated group, those with high school education and no formal education were more likely to experience high emotional barriers (Table 3).

**Table 3:** Multivariable logistic regression analyses of relationship between education and perceived barriers, benefits, and intention

	**Perceived Barriers (Emotional)**	**Perceived Barriers (Practical)**	**Perceived Benefits**	**Intention (Definitely)**
	**OR [95% CI]**	**OR [95% CI]**	**OR [95% CI]**	**OR [95% CI]**
Education				
No formal education	2.29 [1.69‐3.10]***	1.91 [1.42‐2.56]***	1.22 [0.92‐1.63]	0.53 [0.38‐0.72]***
High school	1.32 [1.09‐1.61]**	1.26 [1.03‐1.54]*	1.22 [1.01‐1.49]*	0.75 [0.59‐0.94]*
University graduate	Reference	Reference	Reference	Reference

Abbreviations: CI, confidence interval; OR, odds ratio.

* *P* < .05;

** *P* < .01;

*** *P* < .001; controlling for study group, age, gender, marital status, ethnicity, and self‐rated health.

### 3.2 Practical barriers

Endorsement of practical barriers was low (Table 2). However, respondents with no formal education were more likely to endorse practical barriers than those with more education. The average practical barriers score was 4.92 (SD = 1.66) of 12, indicating a low level of agreement. Respondents with no formal education were more likely to experience a high level of practical barriers (59.0%) than those with high school or equivalent education (48.4%) and a university‐level education (42.3%) (χ^2^[2] = 22.82, *P* < .001). In a multivariable model, respondents with no formal education and high school education were more likely to experience practical barriers than university graduates (Table 3).

### 3.3 Perceived benefits

There was strong agreement with the perceived benefits of screening, with over 90% agreement for all items (Table 2). The perceived benefit items did not consistently follow the expected education gradient. The average score on the perceived benefits scale was 16.50 (SD = 2.31) of 20, indicating strong agreement. In multivariable analyses, respondents with high school education were more likely than university graduates to report a high level of perceived benefits (Table 3).

### 3.4 Intention

Few respondents indicated they would definitely not (0.8%) or probably not (1.7%) take part in CRC screening if they were invited. Approximately one‐quarter (23.4%) indicated they would probably participate, and 74.1% reported they would definitely do the test. Responses were dichotomized to compare “yes, definitely” responses with other responses. Compared with the no formal education group (65.9%), the high school (73.4%) and university graduate (79.1%) education groups were more likely to indicate they would definitely take part in CRC screening (χ^2^[2] = 19.67, *P* < .001) (Table 2). In multivariable logistic regression analyses, compared with the university graduates, respondents with no formal education (OR, 0.53; 95% confidence interval [CI], 0.38‐0.73; *P* < .001) and high school education (OR, 0.75; 95% CI, 0.59‐0.94; *P* = .014) were less likely to report that they would definitely take part in CRC screening (Table 3).

Barriers and benefits were significantly associated with screening intention (barriers‐emotional: *r*'s = −0.30, *P* < .001; barriers‐practical: *r*'s = −0.31, *P* < .001; and benefits: *r*'s = 0.41, *P* < .001). Emotional and practical barriers were associated with each other (*r*'s = 0.59, *P* < .001), and both were associated with perceived benefits (*r*'s = −0.29, *P* < .001; *r*'s = −0.38, *P* < .001, respectively). In a multivariable model controlling for participant characteristics, and perceived barriers and benefits, the likelihood of “definitely” intending to take part in CRC screening was lower among respondents with no formal education and high school education. Respondents with low perceived emotional barriers (OR, 1.86; 95% CI, 1.42‐2.45, *P* < .001), low practical barriers (OR, 1.77; 95% CI, 1.36‐2.31; *P* < .001), and high perceived benefits (OR, 5.18; 95% CI, 3.92‐6.83, *P* < .001) were more likely to definitely intend to take part.

## 4 Conclusions

In this large analysis of UK adults approaching the CRC screening age, we demonstrated a consistent and graded effect of education on perceived emotional and practical barriers toward gFOBt. In turn, people who more strongly endorsed barriers toward CRC screening had weaker intentions to participate. People who perceived high benefits in CRC screening were over 5 times more likely to hold a strong intention to take part; however, no educational gradient was observed for these items, and the majority of people (>90%) endorsed these advantages. Enhancing the perceived benefits of CRC screening may be the most appropriate target for increasing uptake overall, but reducing practical and emotional barriers could have the concomitant effect of reducing educational disparities in CRC screening participation.

Specific barriers were more graded by education than others, suggesting potential targets for reducing educational disparities in screening behavior. There was a noticeable gradient in agreement by education for the emotional barrier, “Doing the FOB test would make me worry more about bowel cancer.” In comparison, fear of an abnormal result was endorsed by over half of the sample, but only a small gradient by education was noted. While a large proportion of the sample were concerned about a negative outcome from screening, more educated individuals may have a greater capacity for self‐regulating their emotions. Understanding how more educated people overcome concerns about test outcome may provide insight into how to support people held back by this fear.

Our data highlight that specific barriers may not be disproportionately endorsed by different educational groups, as previously thought. Studies have suggested disgust may be a barrier to screening uptake.^26^ Dolan and colleagues^13^ noted that people with lower literacy skills were more than twice as likely to be concerned that FOBt screening was “messy.” While a number of people endorsed the “disgust” item in our survey, we noted a small gradient by education in the opposite direction. Interventions aimed at reducing this visceral response (eg, the provision of gloves) may improve overall uptake, but they are unlikely to reduce educational disparities in screening uptake.

Providing accurate and comprehensible information can educate the public about screening and thereby improve their capacity to make an informed choice.^12^, ^27^ Cancer communication can also reduce perceived barriers to screening, by either correcting previous biases or providing accurate information on an unfamiliar topic.^24^, ^28^ European Union guidelines recommend organized screening programs should provide written information to improve public understanding of the aims, benefits, and disadvantages of screening.^29^ However, our data suggest that following exposure to such information, people with lower educational attainment perceived more disadvantages and were less interested in taking part than their more educated counterparts. A mismatch has been noted between the educational skill level of the population and the readability of screening information.^27^ Screening programs should ensure that people with lower educational attainment are not disadvantaged by communication materials.

A recent analysis evaluated 4 attempts to improve the accessibility of the invitation materials used within the NHS BCSP, with a specific focus on reducing inequalities in uptake.^30^ Despite extensive testing processes and use of large cluster‐randomized trials (total n = 747 856), only 1 of 4 interventions marginally reduced disparities. One alternative approach that can reduce disparities in CRC screening participation is patient navigation,^31^ a method involving a trained health professional offering one‐to‐one support to address barriers to screening. A patient navigation trial is planned to promote uptake of flexible sigmoidoscopy in the UK screening programme (McGregor et al, submitted).

The most serious limitations were our poor response rate, and the biased characteristics of responders. The response rate was lower than that of similar studies,^28^, ^32^ and the extent to which our findings generalize beyond the sample is uncertain. The lower response from people living in deprived neighborhoods suggests that the less educated group may be underrepresented. Although we attempted to ensure that the questionnaire was comprehensible to the population, it may have been less accessible to those with less education, leading us to underestimate the prevalence of barriers in this population. Similar concerns about generalizability of the sample are noted because of the strong enthusiasm for being screened; over 97% of respondents reported an intention to be screened. However, there is strong enthusiasm for screening within the general population, and our figures are only marginally higher than a nationally representative UK study.^33^ These data were cross‐sectional, which limits inferences of causality. Approximately half (51.7%) of the sample received additional information materials as part of their invitation pack, which may have biased responses, although study arm was controlled in analyses. Our use of education to investigate inequalities did not encompass other factors that contribute to socioeconomic status.^20^ Understanding associations between other measures of socioeconomic status and screening uptake remains a priority.

Although perceived barriers and benefits were associated with screening intention, we do not know whether these perceptions were appropriately informed by adequate knowledge. Furthermore, without a measure of knowledge and screening behavior, we are unable to comment on whether the less educated respondents were making an informed choice about screening participation. Screening behavior was not recorded because these individuals had yet to be invited to screening. The advantage of this was participant responses were not biased by past behavior,^34^ but the topic of screening may have been less salient to this age group.^35^ Although intention is strongly related to screening behavior, a significant proportion of people fail to act on their intentions.^36^ Our lack of behavioral data prevents us from understanding the psychological constructs related to the “intention‐behavior gap.”

In conclusion, this analysis contributes to a growing literature identifying the educational gradient in psychological constructs known to affect screening decision making. We used a large UK data set of adults approaching CRC screening age to demonstrate that people with lower educational attainment were consistently more likely to report emotional and practical barriers to screening and be less interested in participating. Addressing the barriers and facilitators most strongly associated with education could be one approach to ensuring informed uptake of screening.

## Funding

This paper summarizes independent research funded by the National Institute for Health Research (NIHR) under its Programme Grants for Applied Research Programme (grant reference number RP‐PG‐0609‐10106 and a Cancer Research UK Postdoctoral Fellowship (C9227/A8933) awarded to Dr Robb. Dr Smith is supported by a Cancer Research UK Postdoctoral Fellowship (C42785/A17965). The views expressed in this article are those of the authors and not necessarily those of the NHS, the NIHR, or the Department of Health.

## Conflict of interest

None declared.

## Supporting information

Data S1. Supporting info item
