# Supplementary material for: Inequalities in cancer screening participation: examining differences in perceived benefits and barriers
Source: Psychooncology. 2016 Jul 14;25(10):1168–74. doi: 10.1002/pon.4195 (PMC5082500; doi:10.1002/pon.4195)
Supplement: Supplementary file 1 — Data S1. Supporting info item [file PON-25-1168-s001.docx]

**Appendix**

Emotional attitudes towards FOBt

‘*Doing the FOB test would be disgusting*’,^1^ ‘*Doing the FOB test would be tempting fate*’,^2^ ‘*I would be embarrassed if others knew I had done the FOB test*’,^3,4^ ‘*Doing the FOB test would make me worry more about bowel cancer*’, and ‘*I would be afraid of getting an abnormal result from my FOB test*.’^5^

Practical attitudes towards FOBt

*‘I would not want to keep small amounts of my stools on a card in the house*’,^1^ ‘*I would not have the privacy to do the FOB test*’,^3^ and ‘*I would be unlikely to have the time to do the FOB test*.’^3^

Screening intention

‘*Imagine you have just turned 60 and have received the bowel screening test kit (FOB test kit) in the post, would you do the test?’*.^6^

References

1. Jones RM, Devers KJ, Kuzel AJ, Woolf SH. Patient-Reported Barriers to Colorectal Cancer Screening: A Mixed-Methods Analysis. *Am J Prev Med*. 2010;38(5):508-516. doi:10.1016/j.amepre.2010.01.021.

2. Robb KA, Power E, Atkin W, Wardle J. Ethnic differences in participation in flexible sigmoidoscopy screening in the UK. *J Med Screen*. 2008;15(3):130-136. doi:10.1258/jms.2008.007112.

3. Rawl S, Champion V, Menon U, Loehrer PJ, Vance GH, Skinner CS. Validation of Scales to Measure Benefits of and Barriers to Colorectal Cancer Screening. *J Psychosoc Oncol*. 2001;19(3-4):47-63. doi:10.1300/J077v19n03_05.

4. McCaffery K, Wardle J, Waller J o. Knowledge, attitudes, and behavioral intentions in relation to the early detection of colorectal cancer in the United Kingdom. *Prev Med*. 2003;36(5):525-535. doi:10.1016/S0091-7435(03)00016-1.

5. Tiro JA, Vernon SW, Hyslop T, Myers RE. Factorial validity and invariance of a survey measuring psychosocial correlates of colorectal cancer screening among African Americans and Caucasians. *Cancer Epidemiol Biomark Prev Publ Am Assoc Cancer Res Cosponsored Am Soc Prev Oncol*. 2005;14(12):2855-2861. doi:10.1158/1055-9965.EPI-05-0217.

6. McCaffery K, Wardle J, Nadel M, Atkin W. Socioeconomic variation in participation in colorectal cancer screening. *J Med Screen*. 2002;9(3):104-108.
